# Antioxidant effects of *Bifidobacterium longum* T37a in mice weight loss and aging model induced by D-galactose

**DOI:** 10.1186/s12866-023-02846-5

**Published:** 2023-04-15

**Authors:** Ya Wang, Jiahui Wang, Hehai Li, Jianlong Lao, Dan Jia, Junlong Liu, Jinming Wang, Jianxun Luo, Guiquan Guan, Hong Yin, Youquan Li

**Affiliations:** 1 Department of Veterinary Medicine, College of Coastal Agricultural Sciences, Guangdong Ocean University, Zhanjiang, 524088 Guangdong China; 2 College of Life Science and Engineering, Lanzhou University of Technology, Lanzhou, 730050 China; 3 State Key Laboratory of Veterinary Etiological Biology/Key Laboratory of Veterinary Parasitology of Gansu Province/Lanzhou Veterinary Research Institute, Chinese Academy of Agricultural Sciences, Lanzhou, 730046 China

**Keywords:** *Bifidobacterium longum*, Antioxidant, Anti-aging

## Abstract

**Background:**

Probiotics can reduce free radical scavenging rate and oxidative damage, and improve activity of crucial antioxidative enzymes in host cells. This study aimed to isolate *Bifidobacterium* spp. from faeces of babies, and investigate the antioxidant effects of the *Bif. longum* T37a in mice weight loss and aging model induced by D-galactose.

**Results:**

T37a have good antioxidant properties in the DPPH assay and anti-lipid peroxidation test. Compared with the model group, T37a low group significantly increased the thymus index and the levels of T-AOC and GSH-Px of mice. T37a high group significantly decreased the spleen and liver index of mice and the levels of MDA in liver, significantly increased in liver HDL-C levels, and decreased LDL-C in liver.

**Conclusions:**

T37a may be an anti-aging and weight-loss probiotics for its antioxidant capacity, and it is necessary to study further the molecular mechanism of T37a as antioxidant.

## Background

Aging is a time-dependent and natural physiological process closely related to oxidative stress and free radicals [1, 2]. Accumulation of free radicals and reactive oxygen species damage macromolecules such as membrane lipids, proteins and DNA, and lead to the development of several diseases, such as cancer and neurodegenerative, inflammatory, and heart diseases [3, 4]. In the context of increasing global ageing, it is particular significant to research and develop some anti-aging interventions [5]. Some genetic alterations improved lifespan in some model organisms [6]. However, the inability of genetic alterations blocked its application in human [7, 8]. Therefore, nongenetic interventions (such as antioxidants) are the focus of current anti-aging research [1, 5]. Probiotics are live microorganisms that confer a health benefit to the host when administered in adequate amounts [9]. In vitro and in vivo studies indicate that probiotics exhibit antioxidant potential [10]. *Bifidobacterium* constitutes a major group of probiotics, known for their significant role in sustaining health and enhancing the quality of life, which includes maintaining the healthy intestinal flora and intestinal homeostasis [11], inhibiting the growth of intestinal pathogenic bacteria [12], controlling blood sugar in diabetic patients [13], reduction of serum cholesterol levels [14], anti-tumor activity [15], improving immune function, and so on [16]. There are 32 species of *Bifidobacterium* in the “Berger’s Manual of Systematic Bacteriology” [17].

Probiotics can provide a good strategy to supply dietary antioxidants, but more studies are needed to evaluate antioxidant properties of probiotics before they can be recommended for antioxidant potential. *Bif. longum* T37a was isolated from healthy female baby faeces in Gansu Province, China. But the antioxidant potential of T37a remain unknown. In this study, we aimed to study the antioxidant potential of T37a and validation of the antioxidant potential in vivo setting.

## Results

### Morphological features

**Fig. 1:**
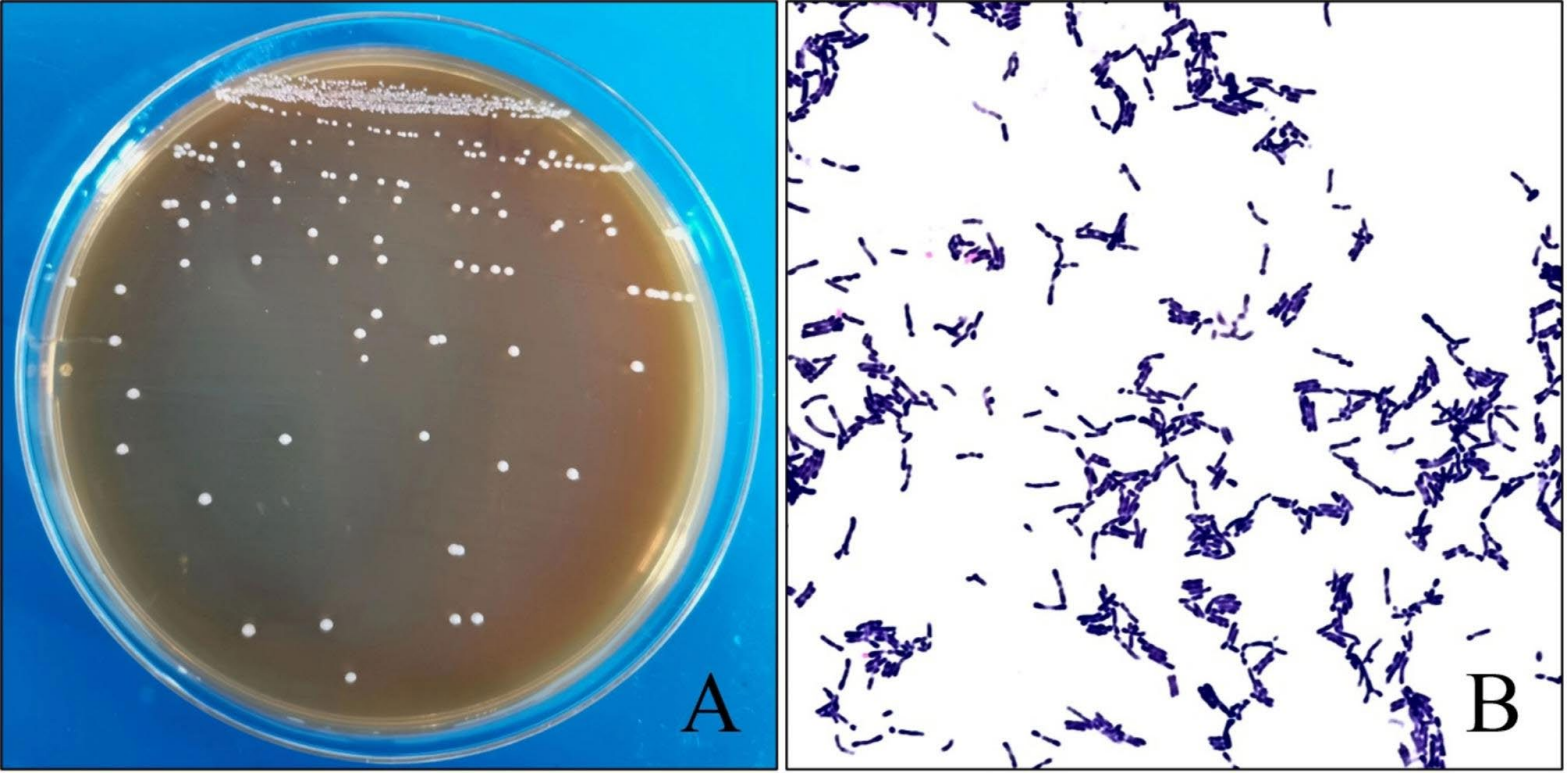
Colony morphology of T37a strain (Fig. 1A) and Gram staining morphology (Fig. 1B)

Two suspected bacterial colonies of *Bifidobacterium* were isolated, labeled T37a and R3. The suspected strain of *Bifidobacterium* was round in BS agar plates with the raised, smooth surface, and white colonies (Fig. 1A). Gram staining showed that they are Gram-Positive Rods, and were polymorphic with V, X, Y or blunt rod-shaped types (Fig. 1B).

### Phylogenetic analysis based on the 16 S rRNA gene sequences

**Fig. 2:**
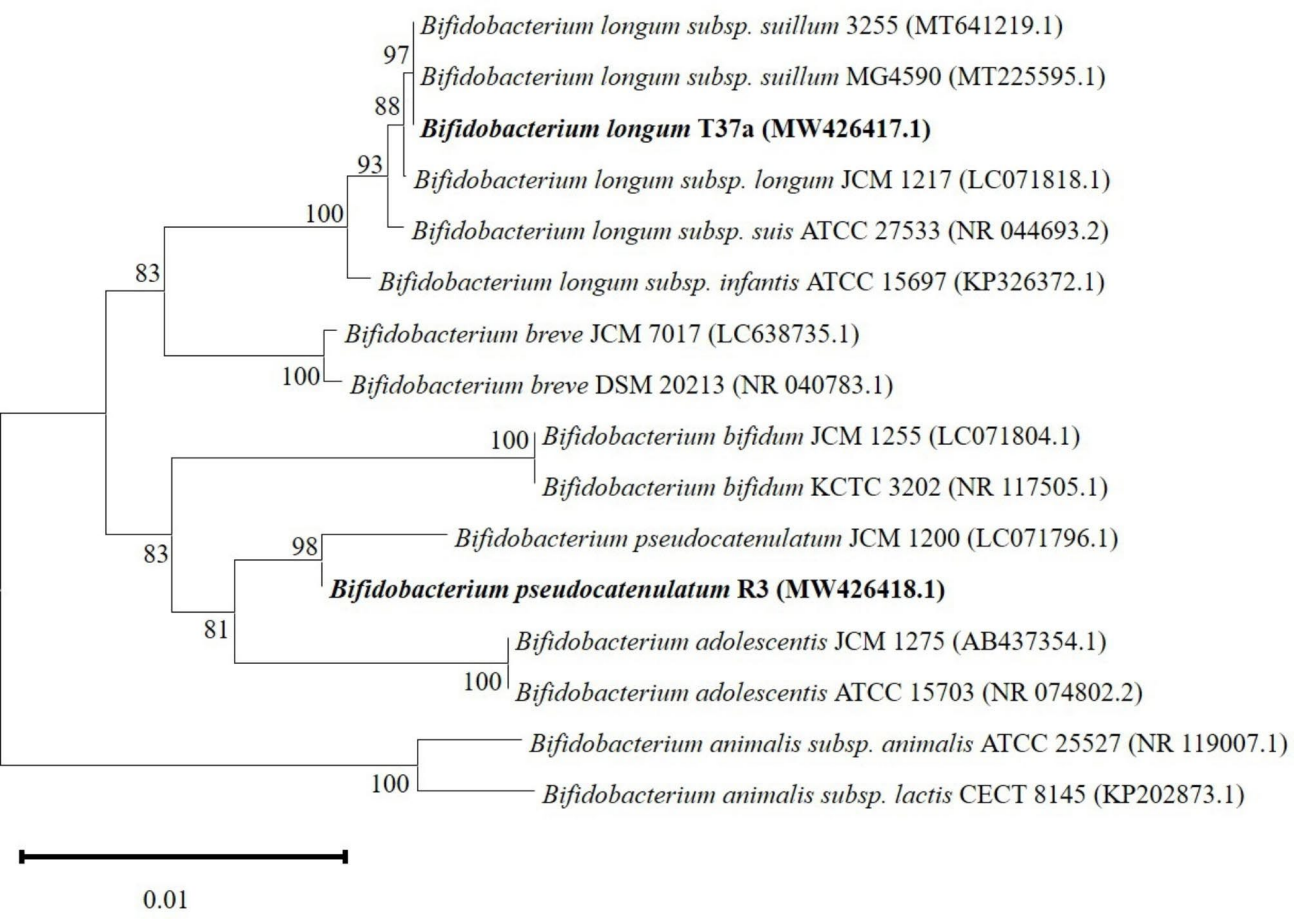
Phylogenetic tree based on 16 S rRNA gene sequences of the strains isolated from different sources. The tree was constructed using the neighbour-joining distance and 1000 bootstrap samples of MEGA program ver. 11.0. *Bif. animalis* subsp. *lactis* is shown as an outgroup

Sequence analysis showed 100% sequence similarities for 16 S rRNA gene between T37a and known *Bif. longum subsp. suillum* strains, including 3255 (100% of sequence identity) and MG4590 (100% of sequence identity); it showed 100% sequence similarities for 16 S rRNA gene between R3 and *Bif. pseudocatenulatum* JCM1200. In addition, T37a, *Bif. longum subsp. suillum* 3255 and *Bif. longum subsp. suillum* MG4590 formed one cluster with an high Bootstrap value (> 95); R3 and *Bif. pseudocatenulatum* JCM1200 formed one cluster in phylogenetic trees with an high Bootstrap value (> 95) (Fig. 2).

### ***In vitro*** antioxidative activity of *Bif. longum* T37a

The antioxidant capacity of the intact cell (IC) and cell-free extract (CFE) of T37a was evaluated. As shown in Table 1, both IC and CFE have DPPH radical scavenging ability, but IC scavenging ability is stronger than CFE. However, anti-lipid peroxidation test had different results, CFE lipid peroxidation ability is stronger than IC.

**Table 1:** Antioxidative activity of *B. longum* T37a in vitro

	Intact-cells (IC)	Cell-free extract (CFE)
DPPH scavenging rate (%)	68.69 ± 4.11	60.9 ± 10.38
Anti-lipid peroxidation rate (%)	40.35 ± 1.32	52.36 ± 1.81

### Effects of ***Bif. longum*** T37a on the weight of aging mice induced by D-galactose

Mice were weighed on day 1, day 15, and day 30. The body weight changes were recorded to evaluate the effects of different groups on mice’s body weight. The results showed that the first 15 days were the stage of establishing an aging model, and the weight of mice in other groups increased significantly (*P* < 0.001 or *P* < 0.0001), compared with the normal group. The next 15 days was the stage of relieving the aging process, except for the normal group, the weight of mice in other groups decrease significantly ( *P* < 0.001 or *P* < 0.0001) (Table 2).

**Table 2:** The body weight changes in mice

Groups	Weight gain in the first 15 days (%)	Weight gain in the next 15 days (%)
**Normal**	39.77 ± 20.69	53.96 ± 11.86
**Control**	99.83 ± 33.83^***^	13.16 ± 6.03^****^
**T37a low**	119.03 ± 46.18^***^	11.40 ± 5.95^****^
**T37a high**	94.45 ± 21.38^****^	12.4 ± 12.01^****^
**VC**	123.35 ± 25.34^****^	20.71 ± 4.58^****^

Note: Compared with control group, * represents P < 0.05; ** represents P < 0.01; *** represents P < 0.001; **** represents P < 0.0001

**Fig. 3:**
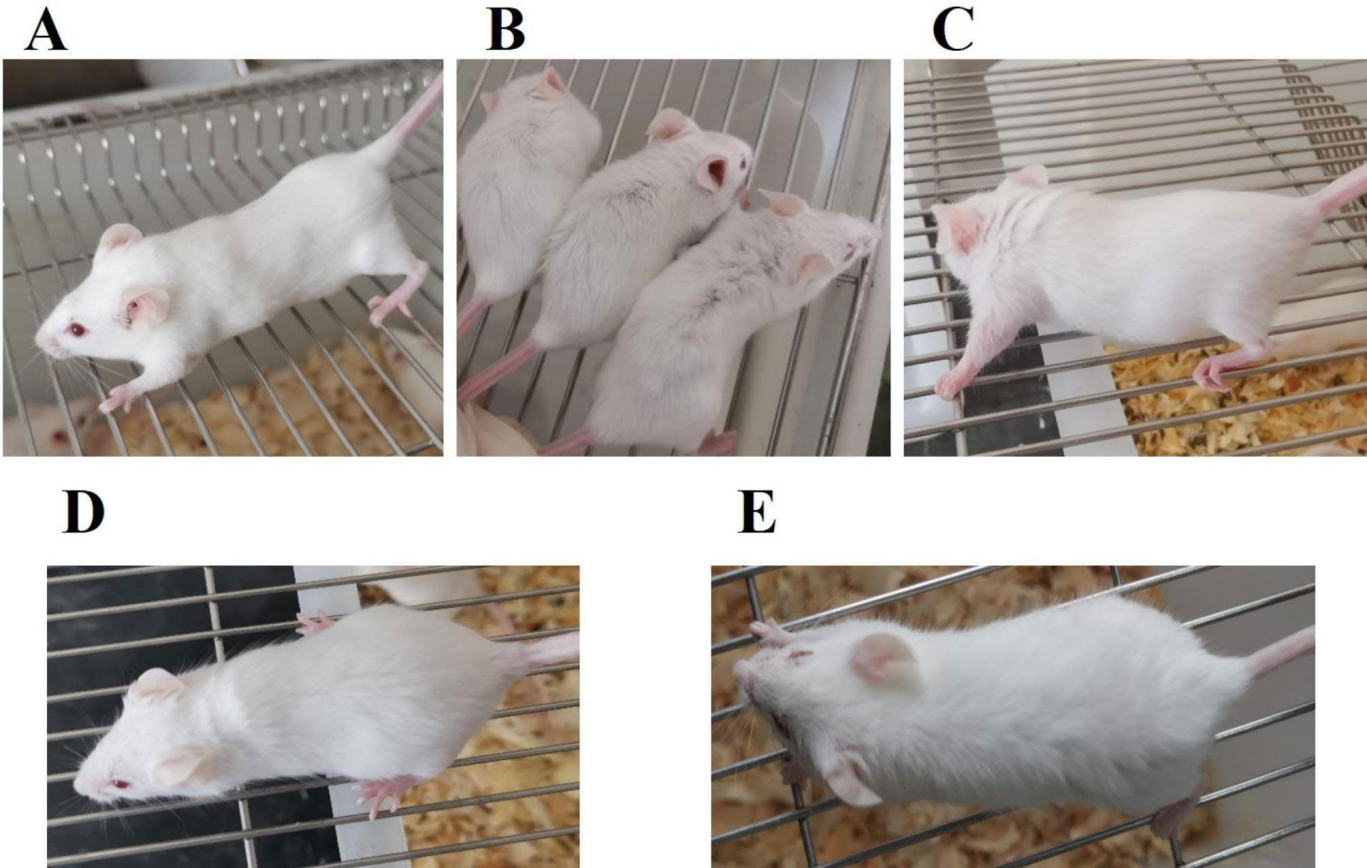
The appearance changes in different groups. (A) Normal group; (B) Control group; (C) T37a low group; (D) T37a high group; (E) VC group. Compared with the control group, mice treated with T37a, and VC exhibited improvements in their hair and spirit

As shown in Fig. 3, there were also differences in appearance among different groups. Mice in the normal group had smooth hair and the mice were mentally active (Fig. 3A), but their furs of mice in the control group were rough and loss, with poor spirit (Fig. 3B). Mice treated with T37a and VC group exhibited improvement in their hair and spirit (Fig. 3C, D and E).

### Effects of ***Bif. longum*** T37a on spleen, thymus and liver indexes of aging mice induced by D-galactose

Compared with the normal group, the injection of D-galactose mice significantly reduced the thymus index (*P* < 0.01). The spleen and liver index were changed but these changes were not significant in the control group. Compared with the control group, T37a could significantly increase the thymus index (*P* < 0.05 or *P* < 0.01) and T37a high group could significantly decrease the spleen and liver index (*P* < 0.05) (Table 3).

**Table 3:** Effects of *B. longum* T37a on spleen, thymus and liver indexes of aging mice induced by D-galactose

Groups	Organ index (g/g) %		
	spleen	thymus	liver
**Normal**	0.357 ± 0.076	0.493 ± 0.125^**^	4.608 ± 0.508
**Control**	0.394 ± 0.082	0.360 ± 0.041	4.367 ± 0.261
**T37a low**	0.383 ± 0.052	0.423 ± 0.055^**^	4.047 ± 0.529^*^
**T37a high**	0.326 ± 0.047^*^	0.405 ± 0.040^*^	3.917 ± 0.478^*^
**VC**	0.371 ± 0.045	0.387 ± 0.059	4.225 ± 0.563

Note: Compared with control group, ^*^ represents *P* < 0.05; ** represents *P* < 0.01

### The effects of ***Bif. longum*** T37a on the levels of T-AOC, SOD, GSH-Px and MDA of mice induced by D-galactose

**Fig. 4:**
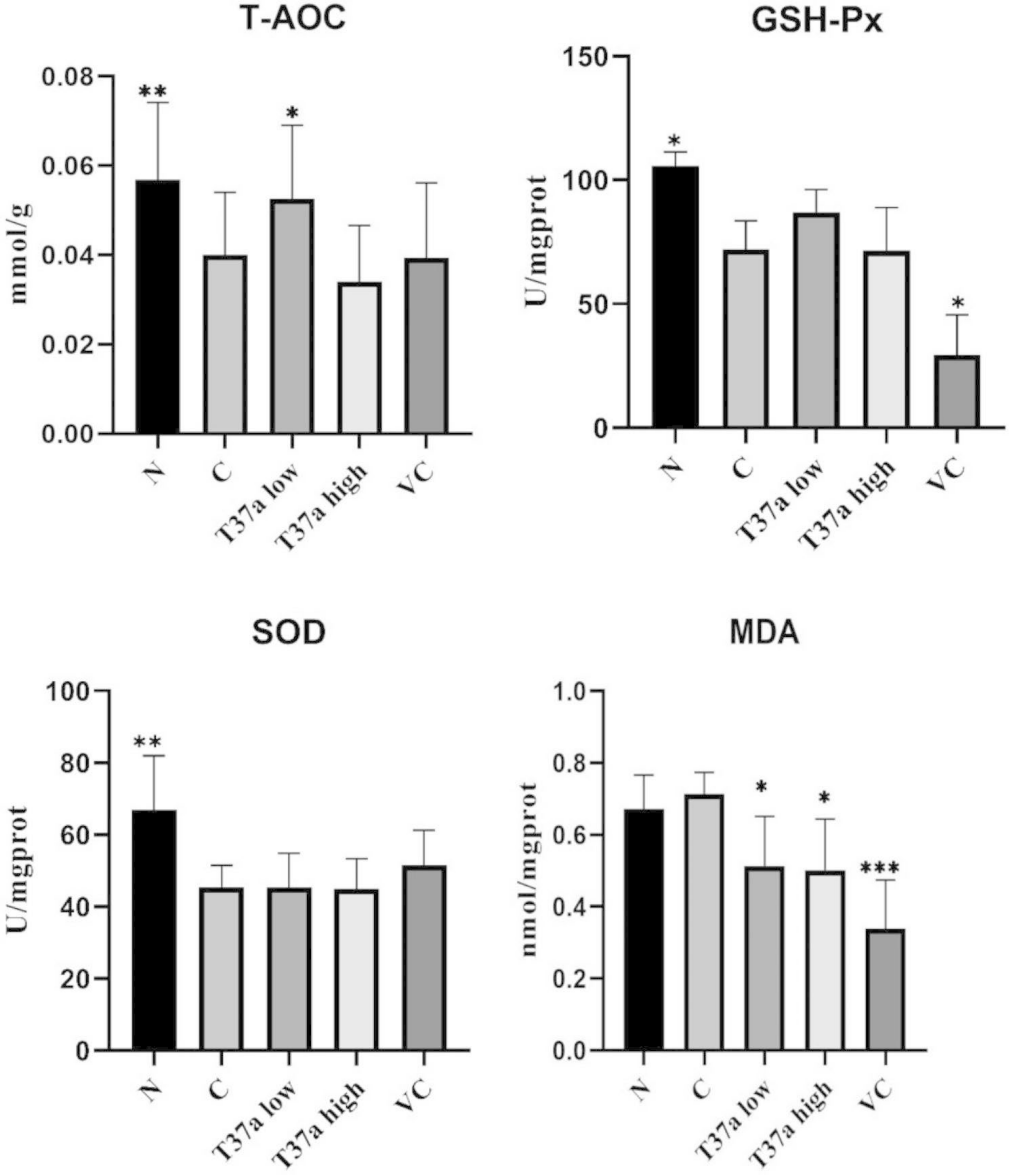
Biochemical indicators in the liver. (A) Effects of T37a on T-AOC; (B) Effects of T37a on the contents of MDA; (C) Effects of T37a on the activities of GSH-Px; (D) Effects of T37a on the activities of SOD. T37a can relieve oxidative stress caused by D-galactose. * represents *P <* 0.05, ** represents *P* < 0.01, *** represents *P* < 0.001 versus the control group

The changes of T-AOC, MAD, GSH-Px and SOD in the liver were presented in Fig. 4. D-gal treatment led to a reduction in T-AOC, GSH-Px and SOD in the liver, compared to the normal group (*P* < 0.05 or *P* < 0.01 or *P* < 0.001), and the results showed that the model was built successfully. T37a low group successfully attenuated the influence of D-gal and significantly increased level of T-AOC and significantly reduced the level of MDA (*P* < 0.05), and the MDA in T37a high group was significantly lower than that in the control group (*P* < 0.05). After the mice supplemented with VC, the level of MDA was significantly reduced (*P* < 0.001), and the content of SOD was significantly increased (*P* < 0.05).

### The effects of ***Bif. longum*** T37a on the levels of HDL-C, LDL-C and TC of aging mice induced by D-galactose

The effects of supplementation with *Bif. longum* T37a on the lipid profile (TC, LDL-C, HDL-C, atherogenic index, HDL-C/TC ratios) are presented in Table 4, and the results demonstrates a causal link between LDL-C concentrations and cardiovascular disease (CVD). D-gal treatment led to a significant reduction in HDL-C and an increase in LDL-C level in the liver compared to the normal group (*P* < 0.01 or *P* < 0.001). T37a high group indicated a significant decrease in LDL-C level compared to the control group by 37.3%, while significant increase in HDL-C level compared to the control group by 70.11%, respectively (*P* < 0.01 or *P* < 0.001). However, in the T37a low group, there were no significant differences in LDL-C and HDL-C, only significant differences in the TC level (*P* < 0.01). Moreover, for the positive control VC group, the improvement levels of HDL-C and TC were lower than the experiment group (*P* < 0.05). D-gal treatment could cause an increase with 248.46% in the liver atherogenic index. While T37a high group showed a significantly decreasing effect and showed significantly less atherogenic index than normal group and the positive control VC group. HDL-C/TC can reflect the degree of health. In general, the larger the ratio, the more healthy it is. D-gal treatment caused a 56.21% decrease than the normal group. Intragastric administration, T37a high showed better characteristics, increased by 43.36% compared to the normal group and 50.75% compared with the VC group.

**Table 4:** The effect of supplementation with *B. longum* T37a on the lipid profile

Group	LDL-C (mmol/gprot)	HDL-C (mmol/gprot)	TC (mmol/gprot)	AI	HDL-C/TC
**Normal**	0.0072 ± 0.0013^**^	0.0201 ± 0.0002^***^	0.0404 ± 0.0052	1.0685	0.4834
**Control**	0.0118 ± 0.0015	0.0081 ± 0.0020	0.0382 ± 0.0071	3.7233	0.2117
**T37a low**	0.0097 ± 0.0028	0.0089 ± 0.0022	0.0486 ± 0.0050^**^	4.4842	0.1823
**T37a high**	0.0074 ± 0.0016^**^	0.0271 ± 0.0061^***^	0.0391 ± 0.0063	0.4429	0.6930
**VC**	0.0088 ± 0.0026	0.0135 ± 0.0011^*^	0.0294 ± 0.0065^*^	1.1752	0.4597

Note: Compared with control group, ^*^ represents *P* < 0.05; ** represents *P* < 0.01; *** represents *P* < 0.001

### The effects of ***Bif. longum*** T37a on histological changes in the liver of aging mice induced by D-galactose

**Fig. 5:**
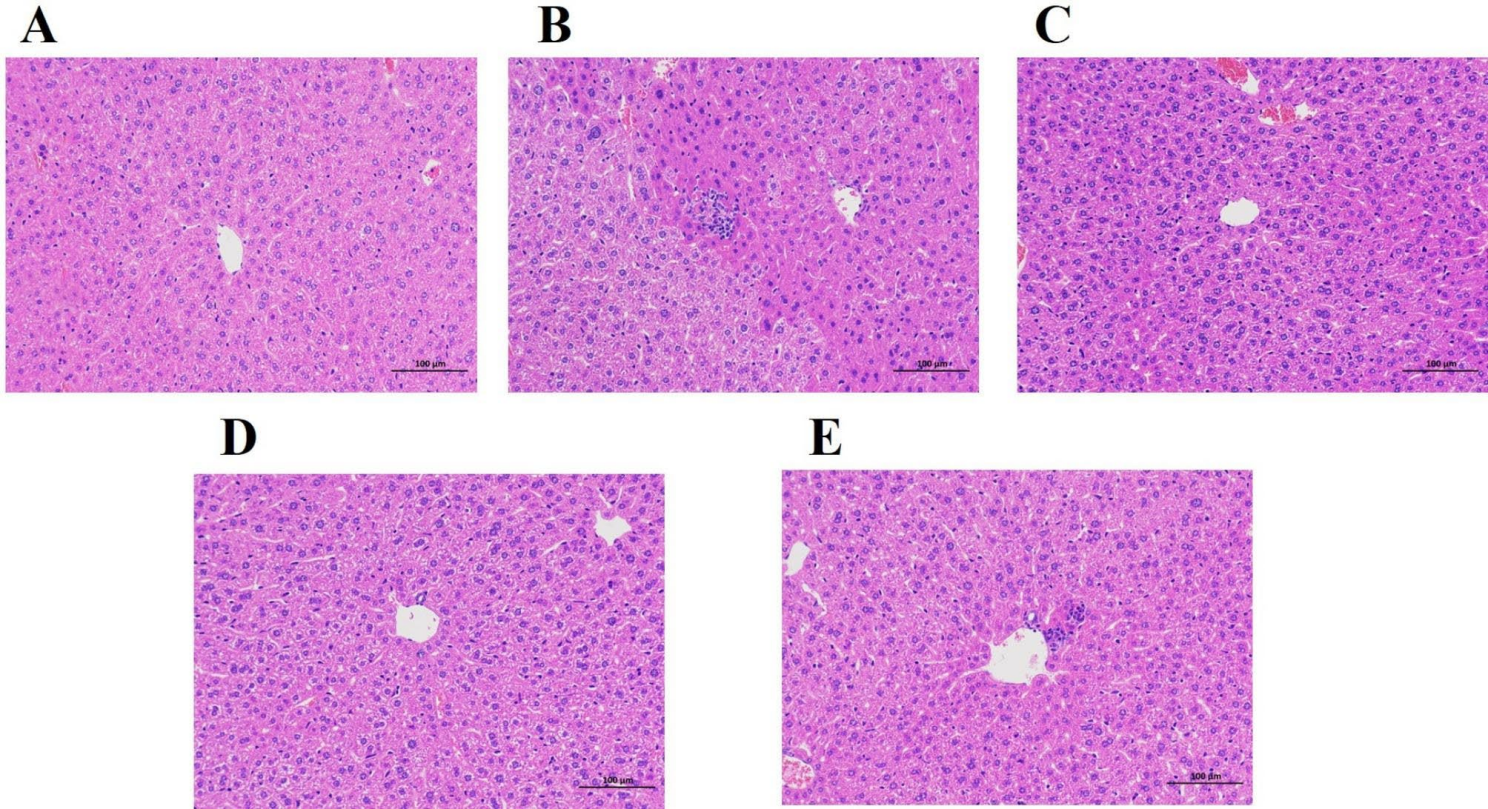
Effects of T37a on the morphology of the liver of aging mice induced by D-galactose(H&E staining, magnification 200×). (A) Normal group; (B) Control group; (C) T37a low group; (D) T37a high group; (E) VC group. D-galactose induced changes in the morphology, and the number of hepatocytes. Meanwhile, the *Bif. longum T37a* had protecting effects on the microstructure of mice viscera

The pathological changes in the liver were shown in Fig. 5. In the normal group (Fig. 5A), HE staining results showed that the central vein was normal, there was no degeneration or necrosis of liver cells, and no inflammatory cell infiltration. Local lymphocyte infiltration, nuclear contraction and eosinophilic enhancement were observed in the control group (Fig. 5B). Mice treated with T37a and VC had exhibited improvements in these situations. In the T37a low group (Fig. 5C) and T37a high group (Fig. 5D), the hepatocyte cords were arranged regularly, with a small amount of inflammatory cell infiltration. In the positive VC group (Fig. 5E), there was a small amount of inflammatory cell infiltration of local. These results indicated that D-galactose induced changes in the morphology, and number of hepatocytes. Meanwhile, the T37a had protecting effects on the microstructure of mice viscera.

## Discussion

Among these aging models, the D-galactose-induced aging model is the most preferred due to its convenience, the least side effects, and higher survival rate throughout the experimental period [18]. The dose of D-gal covered a wide range, and the modelling period is usually different. In this study, the aging model was established by 250 mg/kg D-gal for 15 days. Injection of D-galactose slowed metabolism in mice and significantly increased their body weight. After supplementing with T37a, the metabolism of the mice was improved, and the growth rate of body weight decreased. On 30 day of the test, the body weight of the mice returned to a normal level, and it is similar to Kong’s results [19]. D-gal is mainly metabolized in the liver, and excess of D-galactose in the body may significantly affect the liver [20]. As antioxidant and free radical scavenger, GSH-Px is an important peroxide decomposing enzyme, and widely present in the body. SOD scavenges superoxide radicals, and MDA is one of the most important products of lipid peroxidation. T-AOC determined the total antioxidant level of various antioxidant substances and antioxidant enzymes. Therefore, GSH-Px, SOD, MDA and T-AOC in liver can be used as indicators to reflect the body’s antioxidant capacity. D-galactose treatment can induce oxidative stress in the liver by increasing MDA and decreasing GSH-Px, SOD, and total antioxidant capacity in liver tissues. In this study, feeding *Bif. longum* T37a can significantly reduce MDA and increase T-AOC, GSH-Px, and SOD levels. Supplementing T37a had an effect of relieving aging of the body. This is similar to the results of Kong et al. [19] and Chen et al. [21]. Probiotics are an active area of research due to their diverse applications in health management. Some studies had shown that *Bif. longum* has a regulatory effect on host health [22, 23]. The DPPH scavenging test identified that T37a showed a good stability in vivo utilization, and the scavenging rate of VC was much higher than T37a, but VC had not satisfactory antioxidant effects in vivo. The reason might be that the instability of VC led not to being fully utilized in vivo, and it was similar to Zhao’s results [24].

The immune organ index is a classic indicator to measure the development of immune organs. The thymus index is a preliminary indicator for estimating the body’s non-specific immune function, and reflect the strength of the non-specific immune system. The decrease of the thymus index indicates a decline in immune function. The spleen plays an important role in the body as the largest peripheral immune organ. The liver is the largest organ in the body, and it plays an extremely important role in the body’s metabolism.Reduction of liver index shows a fall of metabolic capacity of animal. In this study, T37a influences these organ indexes, including thymus index, spleen index and liver index, but the difference is not significant. Suo et al. identified that *Lactobacillus paracasei* YBJ01 can improve the index of heart, liver, spleen and kidney organs in D-galactose aging model mice, but the difference is also not significant [25], which is similar to our results. Based on the pathological changes in liver, we found that T37a could improve liver cell morphology and reduced inflammatory cell infiltration.

Although cholesterol is an essential substance for cell function and integrity, long-term elevation of blood cholesterol levels may cause atherosclerosis, and it is considered to be the main risk of cardiovascular diseases (CVD). Many studies showed that probiotics could lower LDL-C and improve HDL-C, as well as lower blood pressure, blood glucose levels, inflammatory mediators and body mass index [26, 27]. In the study, by feeding T37a high dose, the level of LDL-C is significantly reduced, the HDL-C is increased, and the atherogenic index is effectively reduced; all of changes is necessary to guarantee the mice health. The similar results were found in other tests which treatment with *Pediococcus pentosaceus* and *Lactobacillus* also reduced in LDL-C and atherogenic index [28, 29].

All results showed T37a could enhance body’s antioxidant capacity, and low-dose has obvious effect on antioxidant indicators, such as T-AOC, GSH-Px, but the high-dose has evident effect on LDL-C and HDL-C in liver and there is a tendency to lose weight. Therefore, we speculated that *Bif. longum T37a* might have a weight-loss function. The feeding of T37a influences organ index and liver histomorphology but not significant, so we inferred that there might be two reasons: (1) The modeling cycle was short, and the effect on mouse organs was not significant, organ lesions were a long-term accumulation process; (2) The dose gradient designed was small and so there were no obvious differences among experimental groups. This is the first report on validation of antioxidant potential of *Bif. longum T37a in vivo.* Therefore, further studies are required not only to understand the mechanisms of T37a beneficially affecting antioxidant capacity, but also to rule out any of their probable negative effects on health.

## Conclusions

In this study, antioxidant effects of *Bif. longum* T37a in mice weight loss and aging model induced by D-galactose were investigated. Feeding *Bif. longum* T37a can significantly reduce MDA and increase T-AOC, GSH-Px, and SOD levels, and T37a influences these organ indexes, including thymus index, spleen index and liver index, but the difference is not significant. By feeding T37a high dose, the level of LDL-C is significantly reduced, the HDL-C is increased, and the atherogenic index is effectively reduced. After supplementing with T37a, the metabolism of the mice was improved, and the growth rate of body weight decreased. All of changes is necessary to guarantee the mice health. *Bif. longum* T37a have the scope to be developed used as an anti-aging and weight-loss probiotics for its antioxidant capacity. However, further studies are required not only to understand the mechanisms by *Bif. longum* T37a may beneficially affect antioxidant capacity, but also to rule out any of their probable negative effects on health.

## Methods

### Isolation of ***Bifidobacteriaum*** strains from infant faeces

The faeces were collected from female two-month healthy breastfeeding infants in Lanzhou, China. Fresh faeces of infants were serially diluted with 1×PBS and streaked on Bismuth Sulfite Agar plates with glass spreading rod. After 24 h anaerobic culture at 37℃, the smooth, milky or white colonies were selected and continuously passaged untill the pure clone bacterium were obtained with Gram staining and microscopic examination.

### PCR amplification and 16 S rRNA sequencing of isolates

A approximately 1.5-kbp fragment of the 16 S rRNA gene of isolates was obtained using universal primers 27 F (5′-AGAGTTTGATCMTGGCTCAG-3′) and 1492R (5′-TACGGYTACCTTGTTACGACTT-3′) [30]. The PCR products were sequenced by Beijing Tsingke Biology Co., Ltd. (Beijing, China). The sequences were deposited at GenBank under the accession number MW426417 (T37a) and MW426418 (R3), respectively.

### Phylogenetic analysis

The 16 S rRNA sequences were aligned via ClustalW using the MEGA11 software package [31, 32]. Sequences were subjected to similarity search analysis using the BLAST algorithm in the NCBI database. Phylogenetic trees were constructed using the neighbor-joining method with MEGA11 based on the *Bifidobacteriaum* 16 S rRNA sequences determined in this study and others obtained from the GenBank database under accession numbers: MW426417 and MW426418 (in this study), MT641219, MT225595, LC071818, NR044693, KP326372, LC638735, NR040783, LC071804, NR117505, LC071796, AB437354, NR074802, NR119007, KP202873. The stability of the groupings was estimated via bootstrap analysis (1,000 replications).

### Measurement of radical scavenging ***in vitro***

#### Preparation of cell-free extracts (CFE), intact-cells (IC)

T37a was cultivated in BS broth under incubation at 37 °C, 24-48 h. For intact cells (IC) preparation, harvested cell pellets were washed thrice with PBS, resuspended in PBS and adjusted to 10^9^ CFU/mL. For intracellular cell-free extracts (CFE) preparation, cells (10^9^ CFU/mL) were subjected to ultrasonic disruption and sonication was performed for 1 - S pulse on / 2 - S pulse off for 15 min in an ice-bath. Cell debris was removed by centrifugation (10,000 × g, 15 min) and the supernatants were used as CFE.

#### Antioxidative activity of ***Bif. longum*** T37a ***in vitro***

DPPH (2, 2-Diphenyl-2-picrylhydrazyl) free radicals scavenging test has been effectively used to evaluate the free radical scavenging capacity of antioxidant drugs in vitro according to Sharma’s method [15]. Briefly, 500 µL of sample was quickly mixed with 500 µL 0.1 mmol/L DPPH and placed in the dark for 30 min. Then the absorbance at 517 nm was measured. Absolute alcohol was used as negative control. The DPPH scavenging rate was determined using K% = [1-(Ai–Aj)/Ac] × 100% (Ai, the absorbance of sample mixed with DPPH. Aj, the absorbance of sample mixed with absolute alcohol. Ac, the absorbance of negative control.) -.

Unbalanced lipid peroxidation can damage the body and is related to the formation of various diseases. Foods containing anti-lipid peroxidation active substances, such as probiotics, can prevent or reduce these negative health effects. Add 0.5 mL of PBS to 1 mL of linoleic acid emulsion, 1 mL of FeSO_4_ (mass fraction of 1%), and 0.5 mL of the sample to be tested. After shaking, placed it in a 37℃ warm water for 90 min, took it out and vortex to mix. Add 0.2 mL of TCA (mass fraction is 4%), 2 mL TBA (mass fraction is 0.8%), cooled in cold water after 30 min in boiling water bath, centrifuged to collect the supernatants to determine its absorbance at 532 nm. It was determined using that lipid peroxidation rate (%) =(A_0_-A)/A_0_ × 100% (A_0_, the absorbance of the control group at 532 nm, and distilled water is used instead of the sample to be tested. A, the sample to be tested at 532 nm.

#### In vivo model

Fifty 20-day old Kunming female mice, were obtained from laboratory animal center of Lanzhou Veterinary Research Institute, CAAS and held at the animal laboratory of LVRI, CAAS. Animal handling and study protocols were conducted in accordance with the Lanzhou Veterinary Research Institute (CAAS) Animal Care and Use Committee guidelines (No. LVRIAEC2019-011). After a week of acclimation, the mice were randomly divided into five groups (10 per group), including a normal group, an aging model group as control group, T37a low group, and T37a high group as test groups, a VC group as positive group. The control group, test group and positive group were subcutaneously (SC) injected with D-galactose 250 mg/kg at neck back to replicate the aging model, while the control group was injected with saline solution of the same dose for 15 consecutive days.

#### Dose administration

Starting from the 16th day of modeling, mice received daily intragastric administration of the same amount of T37a at doses of 1 × 10^8^ CFU/mL, 1 × 10^9^ CFU/mL, VC at a dose of 500 mg/kg, while the normal and control groups received intragastric administration of normal saline (without water limitation, 30 consecutive days). During this period, the animals were housed in a specific pathogen free (SPF) facility with an internal environmental temperature and relative humidity adjusted to 22 ± 1℃ and 50 ± 10%, respectively, under a 12-h light-dark cycle. The animals were allowed ad libitum access to water and a feed based on corn, soybean, and wheat. Each of the animals was anesthetized with 0.3% pentobarbital sodium (0.15 ml /10 g bodyweight) followed by blood sampling from the mice eyeballs, then humanely euthanized by cervical dislocation, and the liver, spleen and thymus of mice were weighed. The liver, spleen and thymus were immersed in liquid nitrogen, grinded and used in the following experiments.

#### Biochemical indexes and histopathological determination

Twenty-four hours after final administration, the mice were weighed. The mice were sacrificed. Liver, spleen and thymus were taken and weighed to calculate organ index. Organ index = [weight of tissue (g)/body weight (g)]×100. The homogenate was prepared in part of the liver. Total antioxidant capacity (T-AOC), glutathione peroxidase (GSH), superoxide dismutase (SOD), malondialdehyde (MAD), total cholesterol (TCHO), low density lipoprotein cholesterol (LDL-C) and high density lipoprotein cholesterol (HDL-C) levels of liver homogenate were measured. The atherogenic index is an index by the international medical community to measure the degree of arteriosclerosis and calculated by following formula. AI= (TC - HDL-C)/HDL-C. The part of the liver included were fixed with 4% formaldehyde, embedded with paraffin, sectioned, stained with hematoxylin-eosin (HE), and morphological changes of each tissue were observed under light microscope.

### Statistical analysis

The statistical details of all experiments and the sample numbers are described in the figure legends of each figure. All data were expresses as means ± standard error of the mean (SEM). For significant comparisons between two groups, unpaired Student’s *t* test was used, and analyzed using SPSS and GraphPad 8.0. Statistical significance was set at *P < 0.05, **P < 0.01, ***P < 0.001, ****P < 0.0001.

## Data Availability

All 16 S sequences obtained in this study were deposited in NCBI with the accession numbers MW426417.
